# Acute-on-Chronic Liver Failure in a 13-Year-Old Patient With Wilson’s Disease Due to Dengue Virus Infection: A Case Report

**DOI:** 10.7759/cureus.105566

**Published:** 2026-03-20

**Authors:** Ernesto Balmaceda Araya, Mariángeles Murillo González, Akira Osawa Pivovarov, Joshua Robles Ruiz, Ian Taylor Roldán

**Affiliations:** 1 School of Medicine, University of Costa Rica, San José, CRI

**Keywords:** acute dengue, acute fulminant liver failure, acute-on-chronic liver failure (aclf), pediatric hepatic disease, wilson's disease

## Abstract

Acute-on-chronic liver failure (ACLF) is characterized by acute hepatic decompensation in patients with chronic liver disease and is a clinical syndrome associated with high short-term mortality. Most commonly, the underlying cause of chronic liver disease is known prior to the acute decompensation, which is precipitated by another factor. We present the case of a 13-year-old male with previously undiagnosed Wilson’s disease (WD) who developed rapidly progressive ACLF precipitated by dengue virus infection, culminating in multiorgan failure and death. This case highlights the diagnostic challenges and poor prognosis associated with WD-related ACLF and underscores the importance of considering underlying chronic liver disease when hepatic dysfunction appears disproportionate to the expected course of dengue infection in endemic areas. We also discuss current definitions of ACLF, the medical literature pertaining to dengue virus infection and liver failure, and ACLF in the context of WD.

## Introduction

Acute-on-chronic liver failure (ACLF) is a distinct clinical syndrome characterized by acute hepatic decompensation in patients with known or previously unrecognized chronic liver disease [1,2]. According to the Asian Pacific Association for the Study of the Liver (APASL), ACLF is defined as an acute hepatic insult manifesting as jaundice (serum bilirubin ≥ 5 mg/dL) and coagulopathy (International Normalized Ratio (INR) ≥ 1.5 or prothrombin activity < 40%), complicated within four weeks by clinical ascites and/or encephalopathy in a patient with previously diagnosed or undiagnosed chronic liver disease [3]. Reported short-term mortality is approximately 33% and 51% at one and three months, respectively. Although most commonly described in adults with cirrhosis secondary to alcohol use or viral hepatitis [4], ACLF also occurs in pediatric and adolescent populations, in whom inherited or autoimmune liver diseases are frequent underlying etiologies [5].

Among these, Wilson’s disease (WD) is a well-recognized cause of chronic liver failure in children and adolescents [6]. It is an autosomal recessive disorder caused by pathogenic variants in the ATP7B gene, resulting in impaired biliary copper excretion and progressive copper accumulation in the liver and other tissues [7]. Fulminant hepatic presentations are more common in younger patients and are associated with high short-term mortality if not promptly recognized and treated [8]. In many cases, chronic liver disease remains clinically silent until a precipitating event leads to acute decompensation.

In endemic regions, dengue virus infection has been reported as a precipitating factor for ACLF in patients with underlying chronic liver disease. It is typically associated with a self-limited febrile illness and mild to moderate transaminase elevation in many tropical and subtropical regions [9,10]. However, severe hepatic involvement, including acute liver failure, has been described in a minority of cases [11-13].

In this case report, we describe a 13-year-old male who presented with dengue virus infection and acute liver failure, culminating in a fatal outcome due to acute hepatic decompensation in the setting of previously undiagnosed WD. This case illustrates dengue infection as a potential precipitating factor for ACLF in patients with clinically silent chronic liver disease. While hepatic involvement in dengue is typically mild, the disproportionate severity and rapid progression of liver failure in this patient highlight the importance of considering underlying metabolic or chronic liver disorders when the clinical course exceeds the expected spectrum of dengue-associated liver injury. In dengue-endemic regions, early recognition of atypical or severe hepatic dysfunction should prompt clinicians to investigate for underlying liver disease, as timely diagnosis may influence prognosis, guide management, and inform consideration for early referral to specialized centers. We also review the current literature on ACLF, dengue-associated liver injury, and WD to contextualize the diagnostic challenges and clinical implications of this presentation.

## Case presentation

A 13-year-old male presented to the emergency department with subjective fever and vomiting. The patient had no relevant travel or past medical history. Symptoms had begun four days prior to the initial consultation. No epidemiological exposure was initially identified at home, in his neighborhood, or at school.

Vital signs showed blood pressure of 107/38 mmHg, temperature 37.8°C, respiratory rate 19 breaths/min, pulse 113 beats/min, and oxygen saturation 97%. The patient was alert and oriented to person, time, and place. Physical examination revealed a maculopapular rash on the chest. Initial laboratory evaluation (not shown) revealed mild thrombocytopenia without other significant abnormalities. A clinical diagnosis of dengue fever without warning signs was made based on the clinical presentation, and the patient was discharged with outpatient monitoring.

Two days later (day 6 of illness), the patient returned to the emergency department following an episode of syncope, changes in skin coloration, and darkened urine. He reported no focal neurological symptoms. Examination showed jaundice of the skin and mucous membranes, along with signs of dehydration.

Initial laboratory results (Table 1) demonstrated hemoconcentration, severe thrombocytopenia, and a normal white blood cell count; acute kidney injury was also noted. Liver tests showed marked hepatocellular injury, along with hyperbilirubinemia. The magnitude of aminotransferase elevation was considered disproportionate to typical dengue-associated hepatitis and raised concern for severe hepatic injury. The patient was admitted for observation, intravenous rehydration, diagnostic evaluation, and monitoring.

**Table 1:** Laboratory results at hospital admission

Test	Value	Reference range
Hemoglobin	19.4 g/dL	13-17
Hematocrit	52 %	37-49
Platelet count	9.000 U/µL	150.000-450.000
INR	1,42	0.9-1.1
Prothrombin activity	65%	70-120%
Creatinine	1.59 mg/dL	0.6-1.3
Blood urea nitrogen	26 mg/dL	7-25
Albumin	3.4 g/dL	3.5-5.7
Total bilirubin	3.0 mg/dL	0.3-1
Direct bilirubin	1.4 mg/dL	0.03-0.18
Indirect bilirubin	1.6 mg/dL	0.5-1
Aspartate aminotransferase	4472 IU/L	13-39
Alanine aminotransferase	2277 IU/L	7-52
Alkaline phosphatase	147 U/L	34-104
Gamma-glutamyl transferase	167 IU/L	9-64

On the following day (day 7 of illness), the patient developed respiratory distress and progressive deterioration in mental status. Vital signs showed a mean arterial pressure of 60 mmHg. He was tachypneic, tachycardic, and disoriented. Generalized skin mottling was observed over the abdomen and thighs. Arterial blood gas analysis demonstrated metabolic acidosis with hyperlactatemia. Point-of-care ultrasound (POCUS) revealed free intraperitoneal fluid. Inotropic support and noninvasive mechanical ventilation were initiated.

Over the following hours, the patient developed worsening respiratory distress, requiring endotracheal intubation and transfer to the intensive care unit. Empiric broad-spectrum antimicrobial therapy with meropenem, vancomycin, and doxycycline was initiated.

Laboratory studies obtained during the ICU stay (Table 2) showed rapid hematologic deterioration, including anemia and leukocytosis, along with elevated acute-phase reactants, worsening acute kidney injury, and increasing bilirubin levels. Additional testing demonstrated markedly elevated serum copper levels and a positive dengue virus PCR result; however, these results became available at a critical stage in the patient’s clinical course, precluding the initiation of disease-specific therapy.

**Table 2:** Laboratory findings during intensive care unit stay

Test	Value	Reference range
Hemoglobin	8.8 g/dL	13-17
Hematocrit	25 %	37-49
Platelet count	70.000 U/µL	150.000-450.000
International Normalized Ratio (INR)	6,14	0.9-1.1
Prothrombin activity	12%	70-120%
White blood cells (WBCs)	35.700 U/µL	4.000-10.000
Neutrophils	24.030 U/µL	2.500-7.500
Lymphocites	7960 U/µL	1.000-4.000
Creatinine	5.83 mg/dL	0.6-1.3
Blood urea nitrogen	41 mg/dL	7-25
Albumin	2.1 g/dL	3.5-5.7
Total bilirubin	4.1 mg/dL	0.3-1
Direct bilirubin	1.9 mg/dL	0.03-0.18
Indirect bilirubin	2.2 mg/dL	0.5-1
Aspartate aminotransferase	245 IU/L	13-39
Alanine aminotransferase	9640 IU/L	7-52
Alkaline phosphatase	816 U/L	34-104
Gamma-glutamyl transferase	189 IU/L	9-64
Other studies ordered		
C-reactive protein	1.87 mg/dL	<0.5
Ceruloplasmin	8 mg/dL	20-60
Serum copper	916.5 µg/dL	70-140
Extractable copper	638.7 µg/dL	<9.7
Serum zinc	355.3 µg/dL	80-120
Dengue virus polymerase chain reaction (PCR)	Detected	

Less than six hours after ICU admission, the patient developed refractory shock with progressive hemodynamic instability. Hydrocortisone and methylene blue were initiated, and the norepinephrine infusion was titrated to rates exceeding 200 cc/h. Despite maximal resuscitative efforts, the patient died.

**Figure 1:**
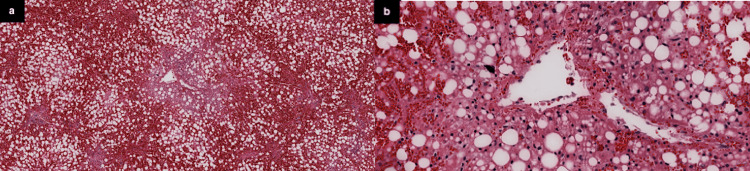
Liver biopsy hematoxylin and eosin (H&E) stain a) Marked sinusoidal congestion and diffuse macrovesicular steatosis. b) (zoomed in) Centrolobular necrosis.

Postmortem liver biopsy with hematoxylin and eosin (H&E) staining (Figure 1) revealed extensive loss of normal hepatic parenchyma, diffuse macrovesicular steatosis, sinusoidal congestion, absence of significant inflammatory infiltrates, and centrolobular necrosis.

**Figure 2:**
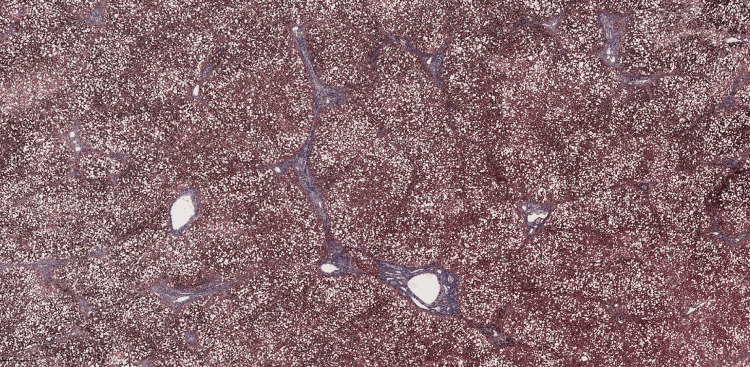
Masson trichrome staining of the liver, showing portal–portal fibrotic bridging

Masson trichrome staining (Figure 2) demonstrated fibrotic bridging between portal tracts. Orcein staining was inconclusive for the presence of copper-associated protein deposits. Quantitative hepatic copper content was 67.85 µg/g of dry weight (reference range 8-40 µg/g in living patients). Although this value was elevated above the normal range, it remained below the classical diagnostic threshold of 250 µg/g dry weight typically associated with Wilson’s disease. However, intermediate hepatic copper levels may occur in acute liver failure due to Wilson’s disease, as extensive hepatocellular necrosis can lead to redistribution of copper and reduced tissue concentrations. Additionally, in pediatric patients or early disease stages, hepatic copper accumulation may be incomplete or unevenly distributed, which may further contribute to lower measured tissue levels. This finding prompted genetic testing for ATP7B gene mutations, which revealed multiple pathogenic variants confirming the diagnosis of Wilson’s disease.

## Discussion

Acute-on-chronic liver failure

ACLF is a severe syndrome characterized by acute hepatic decompensation and failure of one or more organ systems (kidney, liver, brain, coagulation, circulation, or respiration), and is associated with high short-term mortality [1,2]. Several medical societies have proposed diagnostic criteria for ACLF. The most widely used include the criteria from the European Association for the Study of the Liver (EASL), CLIF-C consortium, the Asian Pacific Association for the Study of the Liver (APASL), and the North American Consortium for the Study of End-Stage Liver Disease (NACSELD) [3,14,15].

In our patient, the diagnosis was established using the EASL CLIF-C criteria, which also provide prognostic stratification. The initial CLIF-C score was 33 points, corresponding to an estimated 22.2% one-year mortality. Within 24 hours, rapid clinical deterioration resulted in an increase of the CLIF-C score to 42 points (39.3% estimated one-year mortality), reflecting early progression to severe ACLF. Prognostic scores such as CLIF-C may help guide clinical decision-making, including early referral for liver transplantation in patients with rapidly worsening organ failure.

Dengue infection and liver failure

Dengue virus infection frequently involves the liver, typically presenting with mild to moderate aminotransferase elevation [9,10]. Multiple mechanisms have been proposed to explain dengue-associated liver injury, including direct viral cytopathic effects on hepatocytes, immune-mediated damage driven by cytokine release, systemic inflammatory response, and hepatic ischemia secondary to shock or microcirculatory dysfunction. These mechanisms may act synergistically and are particularly deleterious in patients with reduced hepatic reserve [11].

Kumarasena et al. identified AST levels exceeding 1000 IU/L as a critical threshold associated with progression to ALF in dengue infection, particularly when accompanied early by hyperbilirubinemia, elevated alkaline phosphatase, and persistent nausea or vomiting [16]. Our patient exhibited several of these high-risk features early in the disease course, including rising bilirubin levels and markedly elevated aminotransferases, supporting dengue infection as a significant contributor to the acute hepatic insult. However, the magnitude and rapid progression of liver failure in this case exceeded what is typically observed in isolated dengue-associated ALF, suggesting the presence of an additional underlying hepatic disorder.

A meta-summary by Juneja et al. reported that although dengue-associated ALF may present with severe hepatitis and encephalopathy, outcomes are generally favorable in patients without underlying chronic liver disease, with many cases responding to supportive management [11]. By contrast, the fulminant progression observed in our patient suggests that dengue infection acted primarily as a precipitating insult in the setting of previously unrecognized chronic liver disease.

A case reported by Paul et al. [12] similarly described dengue infection precipitating ACLF in a patient with previously undiagnosed alcoholic liver disease, emphasizing that dengue may act as a trigger for ACLF in endemic regions. Unlike that patient, who recovered with conservative management, our patient developed rapidly progressive multiorgan failure, highlighting the potential severity of dengue-associated hepatic injury when combined with underlying metabolic liver disease such as WD.

Wilson's disease and acute-on-chronic liver failure

This patient’s abrupt clinical deterioration and fatal outcome illustrate the aggressive clinical course that may occur when ACLF develops in the setting of WD, particularly in pediatric patients. WD is a well-recognized cause of ACLF in children and adolescents [17]. Devarbhavi et al. analyzed 68 patients with WD presenting with ACLF and reported that 80% were younger than 18 years, with a mean age of 14.4 years, closely reflecting the age of our patient [8]. Moreover, identifiable precipitants were present in only 11.7% of cases, suggesting that triggering events are frequently absent or unrecognized.

The clinical course observed in our patient is consistent with the high-mortality phenotype described in WD-related ACLF. Encephalopathy has been identified as the only independent predictor of death in this population [8]. In our case, deterioration in mental status was followed by rapid progression to circulatory collapse, respiratory failure, and multiorgan dysfunction, culminating in death within six hours of ICU admission. These findings emphasize the narrow therapeutic window once ACLF becomes established in WD.

Management options for ACLF remain limited. Supportive care in the intensive care unit is essential, but liver transplantation remains the only definitive treatment [5]. Early identification of patients with rapidly worsening ACLF is therefore critical, as prognostic tools such as the CLIF-C score may help identify candidates who could benefit from urgent transplant evaluation. Although post-transplant outcomes are generally favorable [18,19], the decision to pursue transplantation must also consider the severity of multiorgan failure and the potential for medical futility in advanced cases [20].

## Conclusions

This case illustrates a catastrophic, albeit rare, clinical scenario in which dengue virus infection precipitated ACLF in an adolescent with previously undiagnosed WD. In dengue-endemic regions, clinicians should maintain a high index of suspicion when transaminase elevations are extreme, bilirubin rises early, or clinical deterioration occurs disproportionately to typical dengue progression. It should prompt evaluation for underlying chronic liver disease, particularly in pediatric and adolescent patients. The rapid progression from initial hepatic injury to encephalopathy, circulatory collapse, renal failure, and death in this case reflects the high mortality associated with WD-related ACLF and the narrow therapeutic window once multiorgan failure develops. 

Early recognition of ACLF may allow timely referral to specialized centers and consideration of urgent liver transplantation, which remains the only definitive therapy. Ultimately, this case highlights the importance of comprehensive diagnostic evaluation in severe dengue-associated hepatitis and highlights the lethal synergy between acute infectious insults and unrecognized chronic liver disease.
